# Staff and physician protection in neurointervention during the coronavirus disease-2019 pandemic: A summary review and recommendations

**DOI:** 10.1177/15910199211035307

**Published:** 2021-10-20

**Authors:** Julian Maingard, Francisco J Mont’Alverne, Ronil Chandra

**Affiliations:** 1 NeuroInterventional Radiology Unit, 2538 Monash Health, Australia; 2 School of Medicine, Deakin University, Australia; 3 Monash Imaging, Monash University, Australia; 4 Interventional Neuroradiology Unit, Neurology Department, 365090 Hospital Geral de Fortaleza, Brazil; 5 Faculty of Medicine, Nursing and Health Sciences, 365090 Monash University, Australia

**Keywords:** Severe acute respiratory distress syndrome coronavirus 2, coronavirus disease-2019, physician protection, staff protection

## Abstract

The coronavirus disease-2019 pandemic, caused by the novel severe acute respiratory distress syndrome coronavirus 2, has to date resulted in an estimated global death toll of more than 1.5 million with more than 69 million reported cases worldwide. It has become increasingly clear that delivery of effective neurointerventional clinical care means maintaining an able and safe workforce in a rapidly changing environment. Staff and physician protection has become increasingly topical and relevant within the angiography suite both in peripheral and cardiac intervention and in neurointervention. The following review outlines the types of personal protective equipment relevant to neurointerventional care, summarises society guidelines and makes recommendations for the provision of safe care to both staff and patients.

## Introduction

The coronavirus disease-2019 (COVID-19) pandemic, caused by the novel severe acute respiratory distress syndrome coronavirus 2 (SARS-CoV-2), has to date resulted in an estimated global death toll of more than 1.5 million with more than 69 million reported cases worldwide^1^ making it the largest and most severe pandemic since the 1918 influenza pandemic. First emerging in Wuhan, Hubei Province, China in December of 2019 this global pandemic has now resulted in a significant increase in hospitalisation and intensive care unit (ICU) admissions worldwide.^2,3^ While a large proportion of cases result in mild coryzal symptoms and flu-like illness the spectrum of disease ranges from asymptomatic carriers to severe pneumonia and multiorgan failure with 14% of patients presenting with severe disease and 5% suffering critical manifestations.^4^ With a mean incubation period of ∼5.1 days (95% CI 4.5–5.8) up to 97.5% of infected patients develop symptoms within 11 days (95% CI 8.2–15.6).^5,6^ Unfortunately, the heterogeneity in symptomology and incubation period has made tracking and quarantining infected individuals increasingly difficult.

Hospital mortality rates range from 15% to 20% but up to 40% among patients requiring ICU admission^4^ although the true case fatality rate is unclear with inconsistent testing and reporting. What is clear is the potential of the disease to rapidly overwhelm modern health care institutions.

As the pandemic progresses and becomes more widespread it has become increasingly clear that delivery of effective neurointerventional clinical care means maintaining an able and safe workforce.^7^ Early data from Europe and the US highlighted that as much as a hospital bed and intensive care bed numbers are important in the delivery of safe care so too are staffing levels that can deliver safe and effective care.^8^ As a result, staff and physician protection has become increasingly topical and relevant within the angiography suite both in peripheral and cardiac intervention and in neurointervention.^9–12^ The aim of this review is to summarise the types of personal protective equipment (PPE) relevant to continued safe neurointerventional care, summarise society guidelines and make recommendations for personal protection and safety in line with local institutional guidelines.

### Neurointervention during COVID

During the pandemic, many institutions have postponed non-urgent elective surgery to help conserve resources necessary to assist in the care of patients with COVID-19. Additionally, a reduction in normal immunological responses following surgery may accelerate viral replication, increasing the risk of severe COVID-19 infection in previously asymptomatic cases with undetected SARS-CoV-2.^13–15^ In previously asymptomatic cases that underwent elective surgery, the rate of more severe infection of COVID-19 has been demonstrated to be as high as 44% with a mortality rate of up to 23.8%.^15,16^ Pulmonary complications occurred in 51.2% with an increased 30-day mortality of up to 38% accounting for 81.7% of all deaths.^16^

Despite the lack of robust evidence, most societies have recommended deferment of elective neurointerventional procedures if there is high use of hospital and ICU capacity to accommodate patients with COVID-19.^9,11,17–21^ In fact, the decision to postpone an elective procedure should take into consideration the risk for the patient whose elective procedure will be delayed as stated by Elective Surgery Acuity Scale used by the American College of Surgeons.^21^ On the other hand, one should also evaluate the potential exposure and increased risk to patients and staff to COVID-19 in addition to the impact on hospital and ICU capacity, burden on associated staff and availability of PPE, medicines and equipment.^22^

Diversely, some neurointerventional procedures are well-established for the treatment of urgent cerebrovascular diseases, whose inclusion and exclusion criteria should not be modified independently from the dynamics of the COVID-19 pandemic.^23,24^ Mechanical thrombectomy (MT) for acute ischemic stroke is the procedure that best illustrates this since it is a time-sensitive therapeutic intervention with a high level of evidence of benefit.^23^

Emerging data during the early phase of the pandemic from New York reported several cases of large vessel occlusion (LVO) as a presenting feature of COVID-19 in young patients with no prior comorbidities, a stark increase in comparison with the preceding 12 months.^25^ Data now demonstrates that in severe COVID-19 a significant proportion of patients, up to 71% in one series,^4^ meet criteria for diffuse intravascular coagulation. Up to 31% of severely ill patients admitted to the ICU have been shown to suffer thrombotic complications including pulmonary emboli, peripheral limb ischaemia and stroke^26^ with single cohort studies reporting stroke in 2%–6% of patients hospitalised with COVID-19. The proposed mechanisms for stroke in COVID-19 include a hypercoagulable state due to a generalised systemic inflammatory response (with elevated c-reactive protein, d-dimer and ferritin), cerebral vasculitis or cardiomyopathy as part of multiorgan failure.^27,28^ Overall, a 7.6 increase in the odds of acute stroke has been demonstrated with COVID-19 compared with seasonal influenza.^29^

The safe and effective delivery of neurointerventional care in pandemic-stricken countries therefore must acknowledge the increased burden on systems of care not only from increased utilisation of health resources due to COVID-19 itself^30^ and an increased risk to staff but also increased presentations of LVO in the setting of severe disease.^27–29,31^

Recently, the Society of Interventional Radiology defined a list of aerosol-generating procedures (AGPs), which included any procedure requiring endotracheal intubation/extubation, ventilatory support, active airway suction or those requiring significant sedation who may require airway rescue,^32^ criteria well matched to the cohort of patients treated in the neurointerventional suite, particularly in the setting of MT for emergent LVO (ELVO)^11^ in which patients may have a limited collateral history or be difficult to assess.

The concept of the ‘Protected Code Stroke’ is outlined by the Scientific Department on Cerebrovascular Diseases of the Brazilian Academy of Neurology, Brazilian Society of Cerebrovascular Diseases and Brazilian Society of Neuroradiology in their recent recommendation paper. Because AGPs such as bag-valve-mask ventilation, high flow oxygenation, drug nebulisation and oropharyngeal and nasal suctioning are highly likely to occur during an acute stroke, routine contact and droplet precaution with routine use of N95 or KN95 during acute stroke management are recommended^20^ highlighting the importance of staff and physician safety during the pandemic.

### Types of PPE

The SARS-CoV-2 virus particle ranges in size from 60 to 140 nm with surface s-spike protein sizes ranging from 9 to 12 nm.^4^ Modes of transmission include direct patient contact, indirect contact from contaminated objects and aerosolised particles.^33^ During AGPs, particle size is the most important determinant of aerosol behaviour with aerosolised particles <5 µm or smaller shown to be able to remain airborne indefinitely under most indoor conditions unless there is continual removal due to air currents or dilution.^34^ Particles of this range can deposit within the lower respiratory tract of humans with large particles of 6–12 µm able to settle within the upper airway of the head and neck. Studies have demonstrated SARS-CoV-2 particles in sampled air of hospitals in China^35^ and the US^36^ with positive polymerase chain reaction tests in 35% of samples from ICU and 12.5% of samples from a general ward.^37^ Data however is limited, and rather emphasis should be placed on reducing aerosol spread in the setting of AGPs performed in the neurointerventional environment.

Face shields, goggles, gloves and waterproof gowns decrease contamination from both direct and indirect contact but do not provide inhalation protection. Surgical masks, particulate filter respirators (PFRs), elastomeric respirators (ERs) and powered air-purifying respirators (PAPRs) provide increased protection from aerosolised particles, respectively, and have seen widespread use during the COVID-19 pandemic.

### PPE Levels

The World Health Organisation and Centre for Disease Control (CDC) outlines minimum requirements for PPE including technical specifications and performance standards. These include surgical masks for both patient and physician, face and eye protection, PFRs and PAPRs, gloves, aprons, gowns (surgical and isolation) and biohazard bags.^38,39^

#### Surgical masks

Surgical masks offer better respiratory protection compared with not wearing one (adjusted OR 0.15, 95% CI 0.07–0.34).^40^ In fact, the routine wearing of a surgical mask by COVID-19 positive or suspect patients, particularly those with acute ischaemic stroke who may be un-assessable, can further aid in physician and staff protection by reducing the generation of aerosolised SARS-CoV-2 particles.^34^ However, lack of adequate filtration and sealing to the wearers face means that there may be little to no protection of standard surgical masks compared with respirators in the setting of AGPs. Studies demonstrate variable efficacy for surgical masks ranging from 6% to 80% relative risk reduction for infection.^41^ Added protection from aerosolised particles can be achieved using a filtered facepiece such as a PFR, ER or PAPR (adjusted OR 0.04, 95% CI 0.004–0.30).^40,42^

#### Particulate Filter Respirators

PFRs offer added protection over standard surgical masks with a UK Study demonstrating up to 100 times or higher filtration compared with two times reduction by standard surgical masks.^43^ As such, for neurointerventional patients with COVID-19 positive status or unknown status their use has become routinely recommended for proceduralists and their assistants performing procedures in the vicinity of high-risk AGPs (such as intubation performed prior to or as airway rescue during stroke thrombectomy), in clinicians caring for patients in whom AGPs will be required or will occur frequently. PFRs must meet certain standards to be validated for routine clinical use with standards varying depending on geographic region as outlined in Table 1.

**Table 1.** Mask type/certification, regional performance standard and filtration efficacy, which have found routine use in the neuroangiography suite.

Region	Mask type/certification	Performance standard	Filtration (%)
USA	N95	NIOSH^a^ 42c FR 84	≥95
Europe	FFP2	EN 149 2001	≥94
China	KN95	GB2626-2006	≥95
Australia/ New Zealand	P2	1716:2012	≥94
Korea	Korea first class	KMOEL 2017 64	≥94
Japan	DS2	JMHLW notification 214, 2018	≥95

^a^ National Institute for Occupational Safety and Health.

Fit testing is vitally important and PFRs are only as efficacious as their fit test. A poorly fitting PFR provides little to no protection from small aerosolised particles which can bypass the worn filter.^34^

Routine use of PFRs beyond 4 h is not recommended due to prolonged soiling and contamination and prolonged use which could result in deleterious effects for neurointerventional staff and physicians including headache, dermatological effects, thermal and pressure stress.^44–47^ In practical terms, this may not be possible particularly for prolonged procedures during complex endovascular coiling of aneurysms or intracranial shunting lesions (i.e. dural arteriovenous fistulas or arteriovenous malformations) in which procedural time may extend well beyond 4 h.

#### Elastomeric Respirators

ERs consist of half face or full face (with added eye protection) reusable synthetic or natural rubber filter masks with replaceable cartridges. Varying filters are available including (1) N, not oil resistant, (2) R, somewhat oil resistant with an 8 h time limit and (3) P, oil-resistant, rarely with time use limitation. Filtration efficiency ranges from 95% to 99% to 100%.^48^ These may provide a useful alternative to PFRs, however, require adequate fit testing, adequate neurointerventional staff training to clean used equipment and prevent excessive contamination after use. ERs are certified by the Occupational Safety and Health Administration and National Institute for Occupational Safety and Health (NIOSH) with CDC Respirator recommendations outlining levels of filtration as an Assigned Protection Factor (APF). Most half-face ERs provide APF of at least 10 meaning that no more than one-tenth of the contaminants to which the worker is exposed will leak into the inside of the mask. Full face ERs range from APF25 to APF50 depending on the type of filter utilised.

#### Powered air-purifying respirators

PAPRs are able to provide added protection to neurointerventional staff and physicians by filtering viral particles using a battery-operated blower to provide clean air through either a tight or loose-fitting hood or helmet. PAPRs consist of a high-efficiency particulate air filter providing greater efficiency over N95 filters. PAPRs consist of a facepiece, hood or helmet with a breathing tube, cartridge with a filter and a blower as per NIOSH standards. Batteries may be mask or belt-mounted. Although there is no evidence for the routine use of PAPR over other compliant respiratory equipment^49^ their use may be considered in those in whom a tight facial seal is not possible or to prevent long-term adverse effects of PFR use during extending patient contact, particularly during complex neurointerventions. Furthermore, user comfort can be considerable due to lower breathing resistance compared with fit-tested PFRs. Appropriate training is needed in appropriate donning to prevent contamination during removal.^38,39^

### Staff training, donning and doffing

Depending on local institutional guidelines, routine PPE use during neurointerventional procedures should be donned depending on patient COVID positive status and risk of aerosilation. The CDC provides guidelines on appropriate donning and doffing for the care of COVID-19 patients. For most emergent neurointervention (i.e. ELVO) where COVID status may be unknown routine use ideally includes a PFR as per regional standards or PAPR depending on local availability and supply.

Wide-spread training and credentialing of neurointerventional staff and physicians in the available types and appropriate use of PPE is of vital importance in preserving the neurointerventional workforce. A buddy system for both donning of PPE including PFR fit testing and doffing to reduce risk of contamination will vary depending on local institutional guidelines and recommendations but should be practised by all neurointerventionists and staff within the neuroangiography suite. Step by step removal of PPE in a safe and controlled manner should be performed with careful attention to adequate hand hygiene. Removal of PFRs and face shields including radiation goggles should be performed last after adequate hand hygiene with care not to touch the forward-facing components to reduce risk of contamination and spread to the wearers face. Reusable equipment such as radiation goggles should be judiciously cleaned using an appropriate cleaning solution such as a chlorine-based product, as these are often reused. Doffing ideally should occur in a well signed designated area and PPE discarded immediately.

### Society guidelines in documented COVID-19 positive status or undocumented COVID-19 status

Several neurointerventional societies provide consensus guidance on the use of PPE during the COVID-19 pandemic in the neuroangiography suite.^9,11,18–20^ Guidelines vary but there is a consistent message in the use of higher-level PPE during the management of COVID-19 positive or COVID-19 suspect patients. Eye protection and PFRs such as an N95 or higher protection is suggested depending on local supply and institutional guidelines. Where available PAPR is preferred but many institutions worldwide have limited if any stock. Society guidance on PPE use is summarised in Table 2.

**Table 2.** Major neurointerventional society guidelines on PPE use.

Society	Guidance
SNIS^9^	N95 or PAPR Boot or show covers, surgical cap, eye protection, full gown, gloves
European Society of Minimally Invasive Neurological Therapy^18^	N95, FFP2, FFP3, PAPR Cap, gown, single/double gloves, goggles face shield
Scientific Department on Cerebrovascular Diseases of the Brazilian Academy of Neurology, Brazilian Society of Cerebrovascular Diseases and Brazilian Society of Neuroradiology^20^	N95 or KN95 Full sleeved gown, eye protection, gloves, head covering
Chinese Federation of Interventional and Therapeutic Neuroradiology, International Society for Neurovascular Diseases^19^	N95 Isolation gowns, protective suits, safety goggles, face shields, shoe covers, boot covers full-face respirators
The Australian and New Zealand Society of Neuroradiology	Refers to SNIS guidelines^9^ N95 or PAPR Boot or show covers, surgical cap, eye protection, full gown, gloves
Society of Vascular and Interventional Neurology^11^	N95 Double glove, face mask, show covers, protective gear

SNIS: Society of Neurointerventional Surgery; PAPR: powered air-purifying respirator.

### Recommendations

#### PPE tiers

PPE tiers have been described in the routine care of COVID-19 positive patients ranging from tier 0 to tier 3.^50^ Tier 0 is for the routine care of all patients who are not COVID-19 positive or suspect during the COVID-19 pandemic and are generally not appropriate in the neurointerventional suite. Tier 1 is standard droplet and contact precautions incorporating a standard surgical mask and protective eyewear. Tiers 2 and 3 are for use in high-risk scenarios of which many emergent neurointerventional procedures fall such as stroke thrombectomy. Tier 2 incorporates droplet and contact precautions with an N95 or higher PFR and protective eyewear for COVID-19 positive or suspect patients. Tier 3 is contact, droplet and aerosol protection with N95 or higher PFR with mandatory face shields for high-risk patients in whom AGPs will be performed. Faceshields can decrease inhalation of aerosolised particles with a median diameter of 8.5 μm by 96% and surface contamination by 97%^51^ and as such are routinely recommended in the neurointerventional suite. However, smaller aerosol particles of up to 3.4 μm may only be reduced by 23%.

For this review, the following recommendations and allocated staff roles outlined in Table 3 could be considered during neurointerventional care in the neuroangiography suite.

**Table 3.** Allocated neurointerventional staff and physician roles and corresponding PPE tiers.

Allocate role	PPE tier	Roles
Neurointerventional physician/proceduralist	Tier 2 or 3	Team leader oversees workflow, performs the procedure
Neurointerventional trainee/fellow	Tier 2 or 3	Equipment preparation before the patient arrives to room, assists or performed procedure
Medical imaging technologist	Tier 2	Overseas case, ideally from a control room outside the suite, ensures strict adherence to COVID-19 workflow and PPE standards
Scrub nurse	Tier 3	Minimal patient contact during the case, equipment preparation as standard
Scout nurse	Tier 2	Obtains equipment from within the room
Extra nurse	Tier 2	Helpful for obtaining equipment from outside of the room, monitoring adherence to workflow and observing donning and doffing of PPE.
Anaesthesiologist	Tier 3	
Anaesthetic nurse	Tier 3	

PPE: personal protection equipment; COVID-19: coronavirus disease-2019.

## Conclusions

As the pandemic progresses, it has come increasingly clear that we must meet a new ‘COVID normal’. While PPE is not a substitute for other precautions to prevent COVID-19 spread they provide a significant barrier to infection in the neurointerventional clinical environment setting. As such, neurointerventional staff and physicians should familiarise themselves with the types of PPE and society and local institutional guidelines to protect themselves and their patients to be able to continue to deliver safe neurointerventional care.
